# Extramedullary hematopoiesis: mesenchymal stromal cells from spleen provide an in vitro niche for myelopoiesis

**DOI:** 10.1007/s11626-022-00693-8

**Published:** 2022-05-31

**Authors:** Sawang Petvises, Vinson Tran, Ying-Ying Hey, Dipti Talaulikar, Terence J. O’Neill, Jonathan Tan, Helen C. O’Neill

**Affiliations:** 1 Department of Medical Technology, Faculty of Allied Health Sciences, Thammasat University, Bangkok, Thailand; 2 Research School of Biology, Australian National University, Canberra, Australia; 3 Clem Jones Centre for Regenerative Medicine, Faculty of Health Sciences and Medicine, Bond University, Gold Coast, QLD 4229 Australia; 4 ANU Medical School, Australian National University, Canberra, Australia; 5 Department of Haematology, Canberra Hospital, Canberra, Australia; 6 Bond Business School, Bond University, Gold Coast, QLD Australia

**Keywords:** Spleen, Myelopoiesis, Stroma, Niche

## Abstract

Murine spleen has been shown to harbour stromal cells that support hematopoiesis with production of myeloid antigen–presenting cells. Similar stromal lines have now been isolated from long-term cultures (LTC) of human spleen. When human progenitor populations from spleen, bone marrow and cord blood were employed as a source of progenitors for co-culture above splenic stromal lines, myelopoiesis was supported. Human splenocytes gave production of predominantly myeloid dendritic-like cells, with minor subsets resembling conventional dendritic cells (cDC) cells, and myeloid or monocyte-derived DC. Human bone marrow progenitors gave rise to myelopoiesis from hematopoietic progenitors, while human cord blood supported limited myelopoiesis from existing myeloid precursors. Transcriptome analysis compared two stromal lines differing in myelopoietic support capacity. Gene profiling revealed both stromal lines to reflect perivascular reticular cells with osteogenic characteristics. However, the 5C6 stroma which failed to support hematopoiesis uniquely expressed several inhibitors of the WNT pathway. Combined data now show that splenic stroma of both human and murine origin provides a mesenchymal stromal cell microenvironment which is WNT pathway–dependent, and which supports in vitro myelopoiesis with production of specific subsets of myeloid and dendritic-like cells.

## Background

Multiple stromal cell types in bone marrow and lymphoid tissues constitute the cellular niche essential for blood cell development (Dorshkind 1990). Within the bone marrow, stromal cells which contribute to hematopoietic niches have been classified generally as perivascular reticular cells. These lie in close proximity to hematopoietic stem/progenitor cells (HSPC), and adjacent to sinusoids (Crane *et al*. 2017). Extramedullary hematopoiesis in spleen has now been attributed to HSPC localised to the red pulp region (Inra *et al*. 2015). Stromal cells in spleen red pulp which support extramedullary hematopoiesis have been identified as mesenchymal PDGFRβ^+^ cells which secrete CXCL12 and KITL, such that they resemble the perivascular/perisinusoidal reticular cells described originally in the bone marrow hematopoietic niche (Inra *et al*. 2015).

To date, very few studies report stromal lines which support in vitro hematopoiesis. Murine fetal liver stromal lines were shown to maintain HSPC in vitro (Wineman *et al*. 1996), and murine splenic stromal cell lines maintain HSPC and also support in vitro myelopoiesis (Despars and O’Neill 2006a; Despars and O’Neill 2006b; Periasamy *et al*. 2009; Periasamy and O’Neill 2013; Periasamy *et al*. 2013; Petvises and O’Neill 2014b)*.* In this lab, evidence that stromal cells in spleen support hematopoiesis evolved from long-term cultures (LTC) of murine spleen which continuously produced myeloid cells reflecting several dendritic cell (DC) types (Quah *et al*. 2004). Further studies showed that these cells arose from HSPC maintained within the stromal microenvironment (Wilson *et al*. 2000). Most studies on the role of splenic stroma in hematopoiesis have involved mice. When murine bone marrow HSPC were co-cultured above selected splenic stromal lines, continuous myelopoiesis ensued, with production of two main populations of antigen-presenting cells (APC) distinguishable by different abilities to activate T lymphocytes (Periasamy *et al*. 2013, 2009). A novel population of dendritic-like ‘L-DC’ was continuously produced and shown to be the majority cell type. This was distinguishable from a transient, minority population resembling conventional DC (cDC)–like cells (Periasamy *et al*. 2009; Periasamy and O’Neill 2013; Periasamy *et al*. 2013). This novel APC was found to be distinct from the commonly known subsets of cDC and plasmacytoid (p)DC through lack of MHC-II expression, high capacity for endocytosis and ability to activate CD8^+^ but not CD4^+^ T cells (Periasamy *et al*. 2009; Periasamy and O’Neill 2013; Periasamy *et al*. 2013). In contrast, cDC-like cells were produced in low numbers for only 2–3 wk of co-culture, to express MHC-II and to activate both CD4^+^ and CD8^+^ T cells (Periasamy and O’Neill 2013; Periasamy *et al*. 2013). An in vivo equivalent subset of L-DC was subsequently characterised in vivo in both murine and in the human spleen (Tan *et al*. 2011; Petvises *et al*. 2015; Hey *et al*. 2016).

Spleen from humans and mice are similar in terms of the representation and function of dendritic and myeloid subsets (Mittag *et al*. 2011; Petvises *et al*. 2015). We have now investigated the human spleen further for the presence of stromal cells similar to those of murine spleen. The human spleen LTC were generated as a ready source of stroma to derive cloned lines. Isolated lines were then tested for capacity to support myelopoiesis when co-cultured with HSPC isolated from spleen, bone marrow and cord blood.

## Materials and methods

### Tissue culture

Cells were cultured at 37 °C in 5% CO_2_ in air in Dulbecco’s modified Eagle’s medium (DMEM) supplemented with 10% fetal calf serum, 5 × 10^−4^ M 2-mercaptoethanol, 10 mM HEPES, 100U/ml penicillin, 100ug/ml streptomycin, 4 g/l glucose, 6 mg/l folic acid, 36 mg/l L-asparagine and 116 mg/l L-asparagine hydrochloric acid (sDMEM). Stromal cell lines were maintained by passage every 4 d by scraping and dissociation of cells through pipetting and transferral to a new flask (Periasamy *et al*. 2009). Stromal lines were discarded after 5 passages from the original cloned stock to ensure the stability of lines.

### Human tissues and cells

Adult human spleen, adult bone marrow and newborn cord blood were obtained with informed consent of patients from the ACT Haematology Research Tissue Bank (Canberra Hospital, Canberra, ACT, Australia). Tissues were collected under formal written approval of the ACT Health Human Research Ethics Committee (Canberra Hospital, ACT, Australia). Ethical approval for the work was obtained from the ACT Health Tissue Bank, under protocols ETH.1.11.014 (cord blood), ETH4/04.193 (human spleen) and ETHLR.15.162 (bone marrow and spleen). Ethical approval for the study was also obtained from the Human Ethics Committee at the Australian National University (ANU: Canberra, ACT, Australia); Protocol 2012/584. Ethical approval for the experimentation on mice was obtained from the Animal Experimentation Ethics Committee at the Australian National University); Protocol #A2013/11.

The human spleen tissue was obtained from three patients undergoing splenectomy for suspected or known immune thrombocytopenia or lymphoma. Spleens selected for the study were shown to have no histological evidence of lymphoma. Spleen tissues were held aseptically on ice and processed within 12 h of removal. Tissues were chopped into small pieces of 0.5–1.0 cm in dimension. Bone marrow samples were obtained from 2 patients. Cell suspensions of spleen and bone marrow were obtained by forcing cells through a fine-mesh sieve into a Petri dish containing sDMEM. Four distinct human cord blood samples were collected in a vial containing citrate–phosphate-dextrose-adenine-1 (CPDA-1) as an anticoagulant and stored on ice. Ten milliliters of cord blood was diluted by the addition of 30 ml sterile PBS/2 mM EDTA. Red blood cells were lysed by hypertonic treatment (Periasamy and O’Neill 2013; Periasamy *et al*. 2013). Cell suspensions were frozen for later separation.

### Purification of human hematopoietic progenitors

Ficoll-Paque density gradient centrifugation was used to enrich hematopoietic progenitors in spleen, bone marrow and cord blood (Jaatinen and Laine 2007). Preparation of lineage (Lin)^−^ subsets employed MACS® magnetic bead cell separation technology according to the manufacturer’s instructions (Miltenyi Biotec, Gladbach, Germany). A cocktail of biotinylated antibodies specific for human hematopoietic lineage cells (Lineage depletion kit: Miltenyi Biotec) (10 μl per 10^7^ cells: supplemented with antibody to hCD11c) was added to cell suspensions and incubated on ice for 10 min. Antibodies were specific for hCD2, hCD3, hCD11c, hCD11b, hCD14, hCD15, hCD16, hCD19, hCD56, hCD123 and hCD235a, and bound all mature hematopoietic cells, including T cells, B cells, natural killer cells, monocytes/macrophages, granulocytes, DC and erythrocytes. MACS® anti-biotin microbeads (Miltenyi Biotec) were used for separation according to the manufacturer’s protocol. Cell separation was achieved by placing the column in a SuperMACS® II Separator (Miltenyi Biotec). The column was then washed so that flow-through cells could be collected. Flow cytometry of Lin^−^ cells was used to confirm the efficiency of depletion of ≥ 95%.

### Human long-term cultures (LTC)

To establish LTC from the human spleen, a suspension of splenocytes was depleted of only red blood cells, plated in 25cm^2^ flasks and left undisturbed for 10 d at 37 °C, 5% CO_2_ in air and 95% humidity. The medium was then changed by removal and replacement of supernatant every 3 to 4 d. Cells were maintained in this flask for at least 7 wk. This procedure was originally developed in mice (O’Neill *et al*. 2004).

### Establishment of co-cultures

Stromal cells were plated for co-culture following dissociation of confluent stromal cultures using 0.25% trypsin/2 mM EDTA and plating at 10^6^ cells/ml. Stromal cell lines were grown as a monolayer to 80–90% confluency. Lin^−^ splenocytes, bone marrow or cord blood cells were then added at 1–5 × 10^4^ cells/ml as an overlay. Co-cultures were held at 37 °C, 5% CO_2_ in air and 97% humidity. Medium changes were performed every 3–4 d by removal of half volume and replacement with sDMEM. At 7-d intervals, non-adherent cells were collected through removal and replacement of supernatant. Cell yield was determined and cell subsets identified through analysis of surface marker expression by antibody staining and flow cytometry.

### Antibody staining

The procedure used to stain cells with multiple fluorochrome-conjugated antibodies has been described in detail previously (Periasamy and O’Neill 2013; Periasamy *et al*. 2013; Petvises and O’Neill 2014b) ‘Fc block’ specific for FcγII/IIIR (eBioscience) was absorbed to cells ahead of antibody to block non-specific Fc receptor binding. Antibodies used to stain human cells were obtained from Biolegend (San Gabriel, CA, USA) and were specific for hCD11c(clone 3.9), hCD11b(ICRF44), HLA-DR(L243), hCD123(6H6), hCD80(2D10), hCD86(IT2.2), hCD16(3G8), hCD14(HCD14), hCD90(5E10), hCD45RB(MEM-55), hCD38(HIT2) and hCD34(561). Dead cell discrimination involved the addition of 1 μg/ml of propidium iodide (PI) to cells prior to flow cytometric analysis.

Flow cytometry was performed on an LSRII FACS machine (Becton Dickinson: Franklin Lakes, NJ, USA). Voltage, parameter and event counts were programmed using BD FACSDIVA software (Becton Dickinson). Single colour controls were used to set compensation. FlowJo® software (Ashland, OR, USA) was used to analyse data. In general, live cells were gated by the absence of PI staining (PI^−^). Live cells were gated on the basis of forward scatter (FSC) and side scatter (SSC). Fluorescence minus one controls (FMOCs), or isotype control antibodies, were used to set gates to distinguish specific antibody binding.

To stain lineage (Lin)^+^ cells for gating during flow cytometry, a cocktail of biotinylated antibodies specific for human hematopoietic lineage cells was prepared. Antibodies were obtained from Biolegend and were specific for hCD3(OKT3), hCD14(HCD14), hCD19(HIB18), hCD56(HCD56) and hCD45RB (MEM-55), binding mature T cells, B cells, natural killer cells, monocytes/macrophages and granulocytes.

### Microscopy

Stroma alone, LTC, or co-cultures, were photographed using a DM IRE2 inverted research microscope (Leica: North Ryde, NSW, Australia) equipped with DFC digital camera (Leica) to obtain phase-contrast photomicrographs. Images were processed using Leica IM software v4.0.

### Transcriptome analysis

Total RNA was isolated from stromal cell lines using the RNeasy mini kit and following the manufacturer’s protocol (Qiagen). Double-stranded cDNA was synthesised in a two-step process. The first strand of cDNA was synthesised using T7-(dT)_24_ primers and SuperScript II reverse transcriptase (Invitrogen Life Technologies: Mount Waverley, VIC, Australia), followed by second-strand cDNA synthesis. Double-stranded DNA was purified using phenol–chloroform using phase-lock gels (Brinkmann Instruments: Westbury, NY, USA). Subsequent procedures were performed by the Biomolecular Resources Facility (JCSMR, ANU). In vitro transcription and biotin labelling were performed using the BioArray High Yield RNA Transcript Labelling Kit (Affymetrix). cRNA was cleaned using RNeasy Spin columns (Qiagen), fragmented and then labelled with biotin. Fragmented and labelled cRNA was hybridized to Human Gene 1.0ST GeneChips® following the manufacturer’s procedure (Affymetrix: Santa Clara, CA, USA). These were washed and stained on the fluidics station (Affymetrix) ahead of scanning and image analysis using a Gene Array Scanner (Affymetrix). Scanned images of GeneChips were processed using Microarray Suite 5.0 software (MAS5.0; Affymetrix).

### Analysis of microarray data

Gene expression data was analysed using Partek to give average signal values and *p* values by Stephen Ohms (Biomolecular Resource Facility: ANU, Canberra, Australia). Microsoft Excel files containing all information on probeset numbers, genes, signal values and *p*-values were prepared for principal components analysis. Data mining on the basis of average signal values was used to determine gene expression associated with known functions or lineages. Heatmap analysis and agglomerative hierarchical clustering using the Lance-Williams dissimilarity formula involved the use of R project (http://www.r-project.org/). Panther pathway analysis was used to classify the protein class of expressed genes (https://pantherdb.org/).

### Statistical analysis

When replicates could be prepared, data are presented as mean ± S. E. for sample size *n*. The Wilcoxon rank-sum test was used to assess significance (*p* ≤ 0.05).

## Results

### Establishment of human spleen long-term cultures

**Figure 1.**
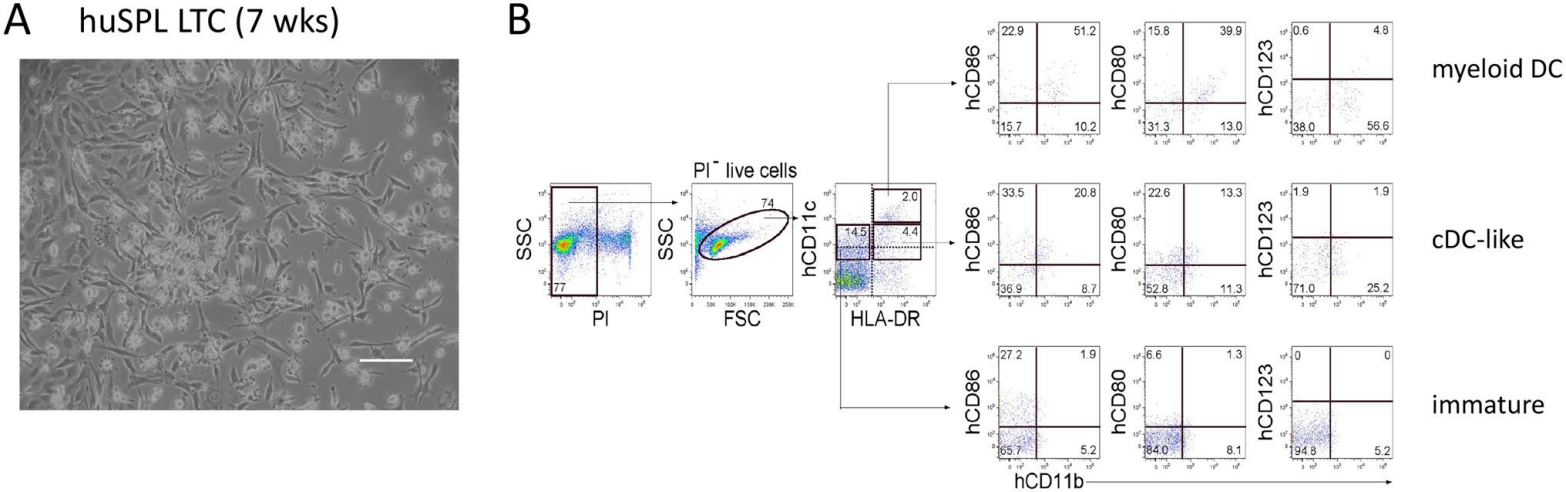
Analysis of myelopoiesis in human long-term cultures (LTC). (***A)*** Whole human spleen cell suspensions were used to establish LTC, shown after 7 wk by phase-contrast microscopy. The bar represents 100μm. (***B)*** Non-adherent cells produced in LTC after 28 days were analysed for production of myeloid and dendritic-like cells. Propidium iodide (PI)^−^FSC^+^ live cells were gated initially. For antibody staining, gates were set on bivariate plots using FMOCs. *Numbers on gates* reflect % cells. Staining for HLA-DR and hCD11c delineated 3 subsets (shown by *rectangular gates*) which were further analysed for marker expression. Cell production shown by flow cytometry is reflective of LTC established from 3 individual human spleens.

Human spleen LTC were established and maintained for at least 7 wk. At biweekly medium change, a 5 ml culture yielded between 10^4^ and 10^5^ non-adherent cells. Both non-adherent cells and fibroblast-like stromal cells were clearly visible after 7 wk (Fig. 1*A*). Non-adherent cells were collected weekly and stained with antibodies for flow cytometric analysis to determine cell production. At 4 wk, three distinct subsets were readily distinguishable through the expression of the hCD11c DC marker and variable HLA-DR expression (Fig. 1*B*). The majority of cells in the hCD11c^hi^HLA-DR^+^ subset expressed hCD86 and hCD11b as well as hCD80, so reflecting myeloid DC. The hCD11c^−/lo^HLA-DR^+^ subset contained cells expressing hCD86, hCD80 and hCD11b, so reflecting cDC-like cells. The hCD11c^−/lo^HLA-DR^−^ subset contained cells expressing hCD86, although not hCD80 or hCD11b. These resemble immature DC, and potentially L-DC (Fig. 1*B*). No plasmacytoid DC (hCD123^+^) were observed (data not shown). A population of hCD11c^−^HLA-DR^−^ cells represents myeloid precursors and any residual lymphoid cells maintained in culture. This result was reflective of many LTC analysed. By comparison with DC subsets produced in murine co-cultures (Periasamy and O’Neill 2013; Periasamy *et al*. 2013; Periasamy *et al*. 2009), cells produced in human co-cultures express lower levels of hCD11c. Lower level staining for hCD11b was also detected, but the CD11b marker is known to be a better marker of the murine than the human DC lineage (Robinson *et al*. 1999; Ziegler-Heitbrock *et al*. 2010).

**Figure 2.**
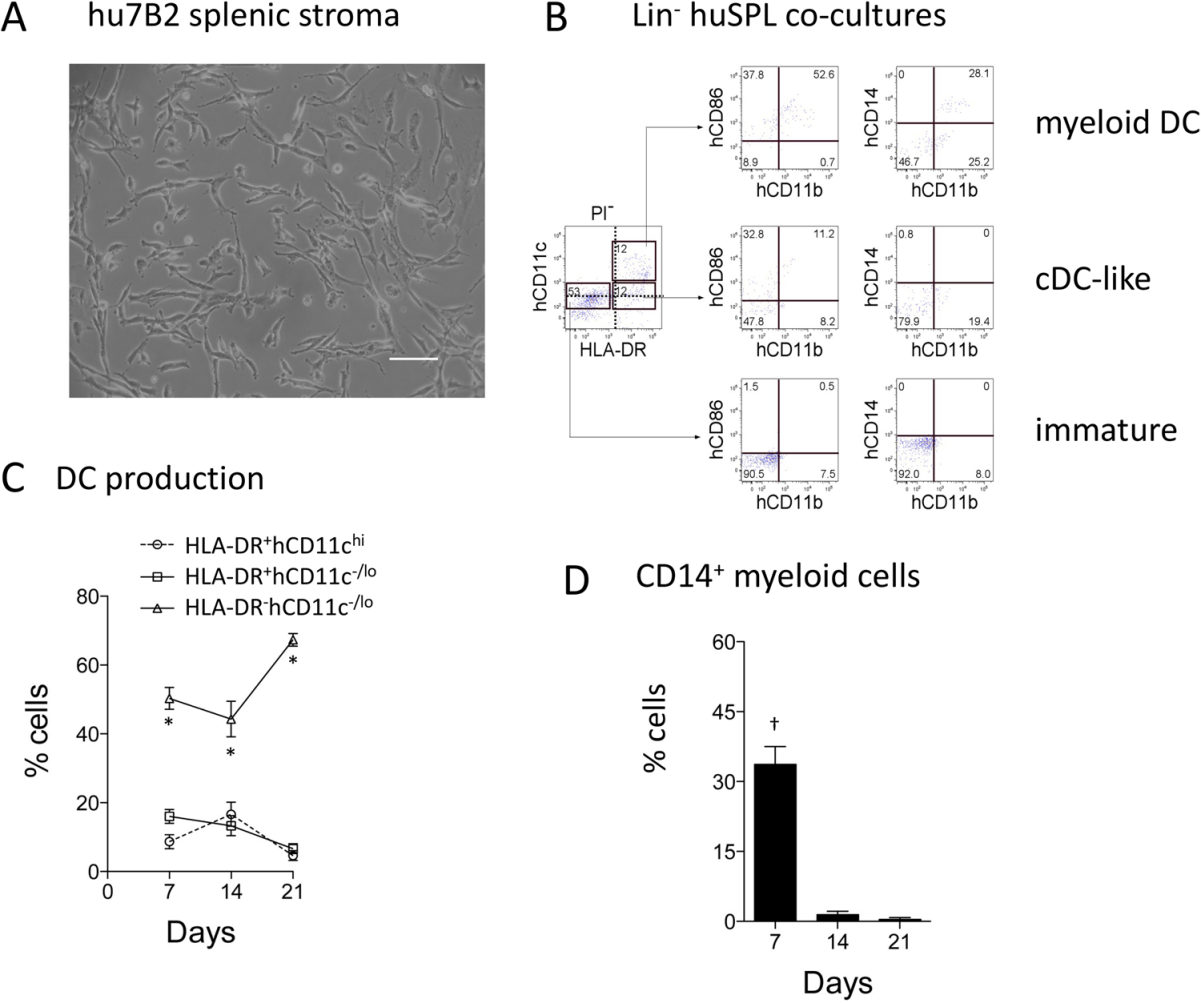
Human spleen co-cultures also support myelopoiesis. Splenic stromal cells were isolated from LTC after 7 wk and established as cloned lines. (***A)*** Phase-contrast microscopy shows the hu7B2 stromal line. The *bar* represents 100 μm. Three independent co-cultures of Lin− human splenocytes from three different spleen donors were established above hu7B2. Cells produced were analysed flow cytometrically at 7, 14 and 21 d. Cultures of splenocytes alone showed no viable cells after 21 d (not shown). (***B)*** Non-adherent cells produced during co-culture were stained with antibodies to delineate myeloid/dendritic-like cell subsets. Flow cytometry of early 7-d co-cultures is shown for one spleen co-culture. PI^−^FSC^+^ live cells were gated initially (as shown in Fig. 1). Staining with CD11c and HLA-DR delineated three subsets indicated by rectangular gates as CD11c^−/lo^ or CD11c^hi^ cells. The expression of hCD86, hCD14 and hCD11b then distinguished cells further. Gates were set on bivariate plots using FMOCs. Numbers on gates reflect % positive cells. (***C)*** Production of subsets was calculated by proportional representation over 21 d of co-culture following multiple medium changes. Data is shown as mean ± S.E. for three distinct co-cultures. Cell production which is significantly different (*p* ≤ 0.05) from all other subsets on the same time point is indicated by an *asterisk*. (***D)*** Transient production of the hCD14^+^ subset of myeloid DC is shown as mean ± S.E. for the three co-cultures. Cell production levels significantly different (p ≤ 0.05) from other time points are shown by a *dagger symbol*.

In order to isolate pure stromal cell lines, non-adherent cells were washed out of LTC, and stromal cells isolated and passaged several times to further deplete any remaining hematopoietic cells. HD248 stroma was then cloned by single-cell sorting and deposition, and clones expanded in culture. Multiple (95) splenic stromal clones were derived from the parent HD248 line and assessed for in vitro growth. These lines, like the hu7B2 line shown in Fig. 2*A*, retain a fibroblastic-like appearance. Stroma could be maintained as a continuous cell line and passaged regularly either by scraping or enzyme dissociation of cells when replicate cultures were needed for experimentation.

### Spleen and bone marrow contain progenitors for DC production

Several stromal lines were selected for their superior growth capacity. To test their ability to support hematopoiesis, stromal lines were plated, and Lin^−^ human splenocytes overlaid above the stroma. Three independent co-cultures were established above the hu7B2 stroma and non-adherent cells collected weekly for flow cytometric analysis. After just 7 d, three subsets of cells could be delineated on the basis of hCD11c and HLA-DR expression (Fig. 2*B*). A hCD11c^hi^HLA-DR^+^ subset contained cells with high expression of hCD86 and hCD11b, with some cells expressing hCD14, so reflecting myeloid or monocyte-derived DC (Katoh *et al*. 2005). A hCD11c^−/lo^HLA-DR^+^ subset contained cells expressing hCD86 but not CD14, and some with hCD11b, so reflecting cDC-like cells. The hCD11c^−/lo^HLA-DR^−^ subset showed some expression of hCD86, such that cells reflect human immature DC and maybe developing L-DC (Fig. 2*B*). The profile of cells produced after only 7 d co-culture using a highly enriched population of hematopoietic progenitors from spleen was reflective of cells produced in 4-wk LTC shown in Fig. 1 established with whole splenocytes. Over 21 d, hCD11c^−/lo^HLA-DR^−^ cells became the majority population produced in co-cultures, consistent with evidence for L-DC as the majority population produced in murine co-cultures over time (Periasamy and O’Neill 2013; Periasamy *et al*. 2013). Subsets of hCD11c^hi^HLA-DR^+^ myeloid and hCD11c^−^/^lo^HLA-DR^−^ cDC-like cells were produced in far lower numbers with time and disappeared after 21 d (Fig. 2*C*). These co-cultures provide evidence that splenic stroma can support myelopoiesis in spleen HSPC. Myeloid cells expressing the hCD14 marker of monocytes or monocyte-derived DC were produced early, but then dropped significantly after 7 d, suggesting transient production of these cells from pre-existing precursors (Fig. 2*D*). Production of a predominant immature L-DC-like population, with transient production of cDC-like cells and monocyte-derived DC, mirrors the outcome of murine spleen co-cultures reported previously (Periasamy and O’Neill 2013; Periasamy *et al*. 2013; Petvises and O’Neill 2014a). Again, subset identification was made more difficult by the low expression of both CD11c and CD11b on human DC subsets.

**Figure 3.**
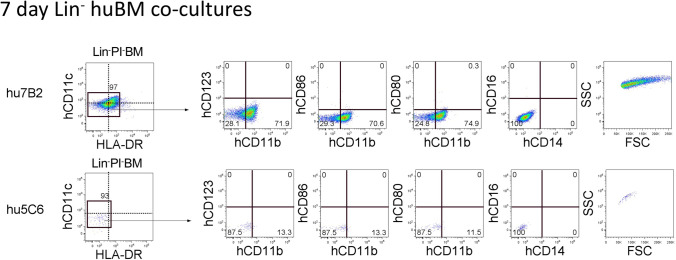
The human bone marrow provides progenitors for myelopoiesis in co-cultures. Co-cultures of Lin^−^ human bone marrow cells (huBM) were established over the cloned hu7B2 and hu5C6 stromal lines. Total non-adherent cells produced after 7 d were collected for quantitation and phenotypic identification through flow cytometry. PI^−^FSC^+^ live cells were gated initially. Gates were set on bivariate plots using FMOCs, and numbers on gates reflect % cells. A single subset of live, large (high FSC) cells (*rectangular gate*) was further assessed for marker expression.

The human bone marrow was then assessed as a source of progenitors for hematopoiesis in human stromal co-cultures. Productive co-cultures were established by the overlay of Lin^−^ human bone marrow containing a highly enriched population of hematopoietic progenitors above the hu7B2 but not the hu5C6 stromal line (Fig. 3). Non-adherent cells were collected and analysed flow cytometrically. The analysis involved gating to detect cells expressing hCD11c and HLA-DR initially. For the hu7B2 stromal co-cultures, analysis as early as 7 d revealed a single population of cells heterogeneous in their expression of HLA-DR, and with very few cells expressing the DC marker CD11c. A majority of cells showed expression of CD11b, but no cells expressed the DC markers hCD80 and hCD86, the myeloid marker hCD14 or the plasmacytoid DC marker hCD123 (Fig. 3). The human stromal line hu7B2 rapidly supports early myelopoiesis within 7 d of co-culture with human bone marrow progenitors. The hu5C6 stromal line was considered a non-supporter of early myelopoiesis, and the hu7B2 line was selected as a good supporter for further gene profiling.

Human stromal lines were also shown to support hematopoiesis in murine bone marrow (data not shown). After only 14 d, each of 6 distinct stromal lines showed outgrowth of a main cell type with a common phenotype as CD11b^hi^CD11c^lo^ cells which did not stain for MHC-II or B220 but were uniformly F4/80^+^ cells. These are reflective of the previously described dendritic-like L-DC population of antigen-presenting cells derived in murine spleen stromal co-cultures (Periasamy and O’Neill 2013; Periasamy *et al* 2013; Petvises and O’Neill. 2014a, b).

### Cord blood as a poor source of hematopoietic progenitors

**Figure 4.**
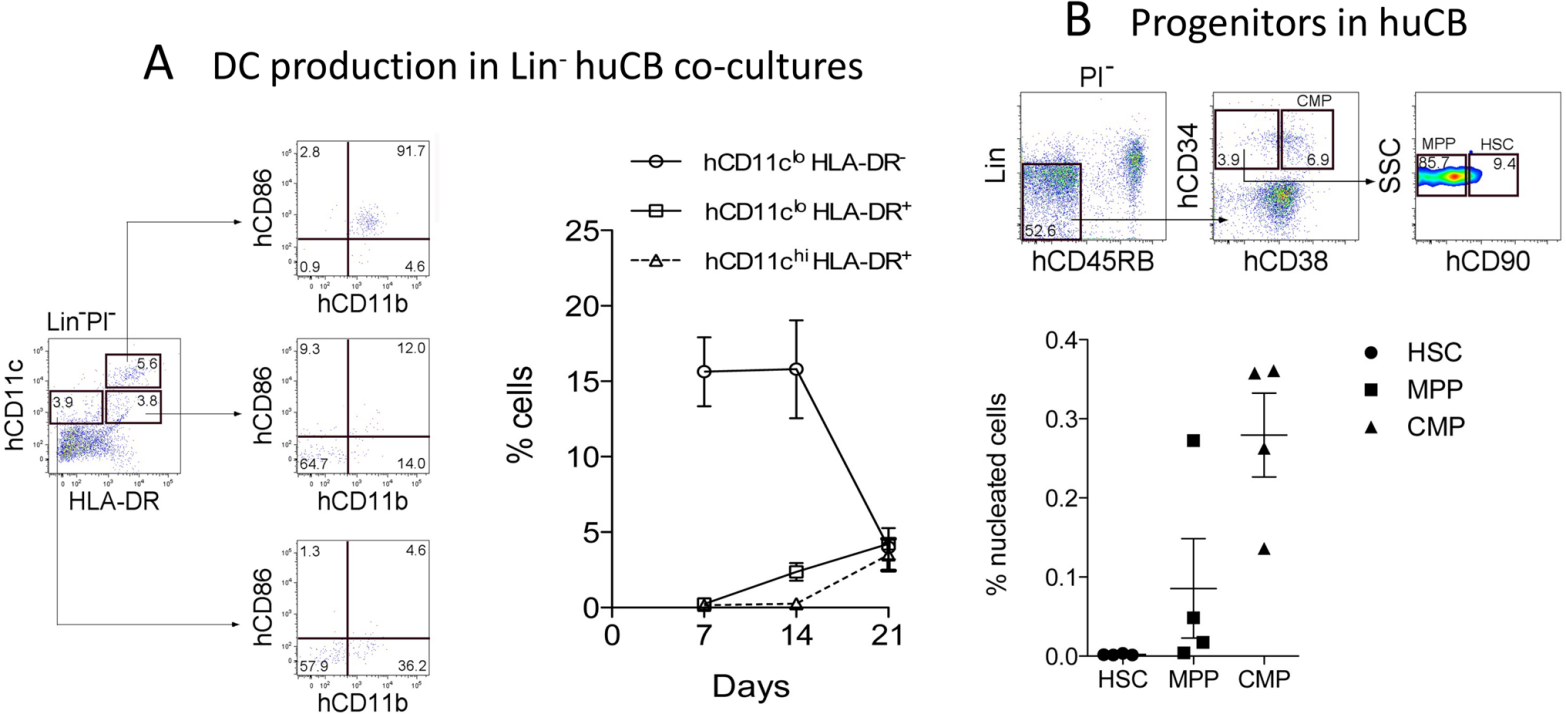
Cord blood is a poor source of progenitors for myelopoiesis supported by stroma. (***A)*** Co-cultures of Lin^−^ human cord blood (huCB) were established above hu7B2 stroma and analysed for production of myeloid and dendritic-like cells. Non-adherent cells were collected at 7, 14 and 21 d. Staining for HLA-DR, hCD11c, hCD11b and hCD86 (shown at 21 days) revealed three subsets as seen in Figs. 1 and 2. The graph shows representation of three subsets produced over 21 d as mean ± S.E. for triplicate experiments. Cell production levels significantly different (p ≤ 0.05) from all other subsets on the same time point are shown by an *asterisk*. (***B)*** The human cord blood samples were stained with antibody and analysed flow cytometrically to identify progenitors present. One representative sample out of four is shown. PI^−^ cells were gated on Lin/CD45RB, and then analysed for progenitor subsets. *Rectangular boxes* delineate hCD34^+^hCD38^+^ cells as common myeloid progenitors (CMP), and hCD34^+^hCD38^−^ cells as either hCD90^−^ multipotential progenitors (MPP) or CD90^+^ hematopoietic stem cells (HSC). Subset representation was calculated, and values shown represent mean ± S.E. for four independent cord blood samples.

In comparison with spleen, cord blood was a poor source of progenitors. Co-cultures were established by the overlay of Lin^−^ cord blood cells above hu7B2 stroma, and cells collected and analysed flow cytometrically at 7-d intervals. As for spleen co-cultures shown in Fig. 2, three main subsets of dendritic-like cells were identifiable (Fig. 4*A*). The hCD11c^lo^HLA-DR^−^ subset which showed no expression of hCD86 with some expression of hCD11b expression reflected immature DC or possibly the L-DC subset described in spleen. Production of these cells reached a peak of ~ 17% of cells after 14 d, although numbers declined to 3.9% by 21 d (Fig. 4*A*). The hCD11c^lo^HLA-DR^+^ subset reflected cDC-like cells with some staining for hCD86 and hCD11b, representing only 4% of cells after 21 d. A subset of hCD11c^hi^HLA-DR^+^ cells expressing hCD86 and hCD11b and reflecting myeloid DC was only detectable at 21 d (Fig. 4*A*). Overall, the human cord blood did not appear to contain progenitors to sustain myelopoiesis in co-cultures past 14–21 d.

The human cord blood from four individuals was assessed further for the content of progenitor types (Fig. 4*B*). Flow cytometric analysis involved initial gating to remove cells expressing the hCD45RB hematopoietic marker as well as lineage markers. The remaining cells were then assessed for hCD34 and hCD38 expression, essential markers for isolation of human HSC (Baum *et al*. 1992; Kondo *et al*. 1997). hCD34 versus hCD38 delineates CMP and upstream progenitors (Majeti *et al*. 2007; Seita and Weissman 2010). The hCD34^+^hCD38^−^ subset can be divided to give hCD90^+^ HSC and hCD90^−^ multipotential progenitors (MPP) (Chao *et al*. 2008; Seita and Weissman 2010; Doulatov *et al*. 2012) (Fig. 4*B*). Replicate cord blood samples were found to be a poor source of HSC, with a higher representation of MPP and common myeloid progenitors (CMP) (Fig. 4*B*). The average number of HSC in the human cord blood was 0.0021% of nucleated cells, while average numbers of MPP and CMP were 0.085% and 0.279%, respectively (Fig. 4*B*). A previous study in mice identified self-renewing HSC as the source of L-DC produced continuously in bone marrow co-cultures over spleen, with a role for MPP in the short-term production of these cells (Petvises and O’Neill 2014b). The absence of HSC in the human cord blood and the low level of MPP are consistent with the inability to sustain myelopoiesis in stromal co-cultures. While CMP are present in higher numbers, their contribution to myelopoiesis in vitro is not supported well by splenic stroma.

### Analysis of human splenic stroma through gene expression analysis

**Figure 5.**
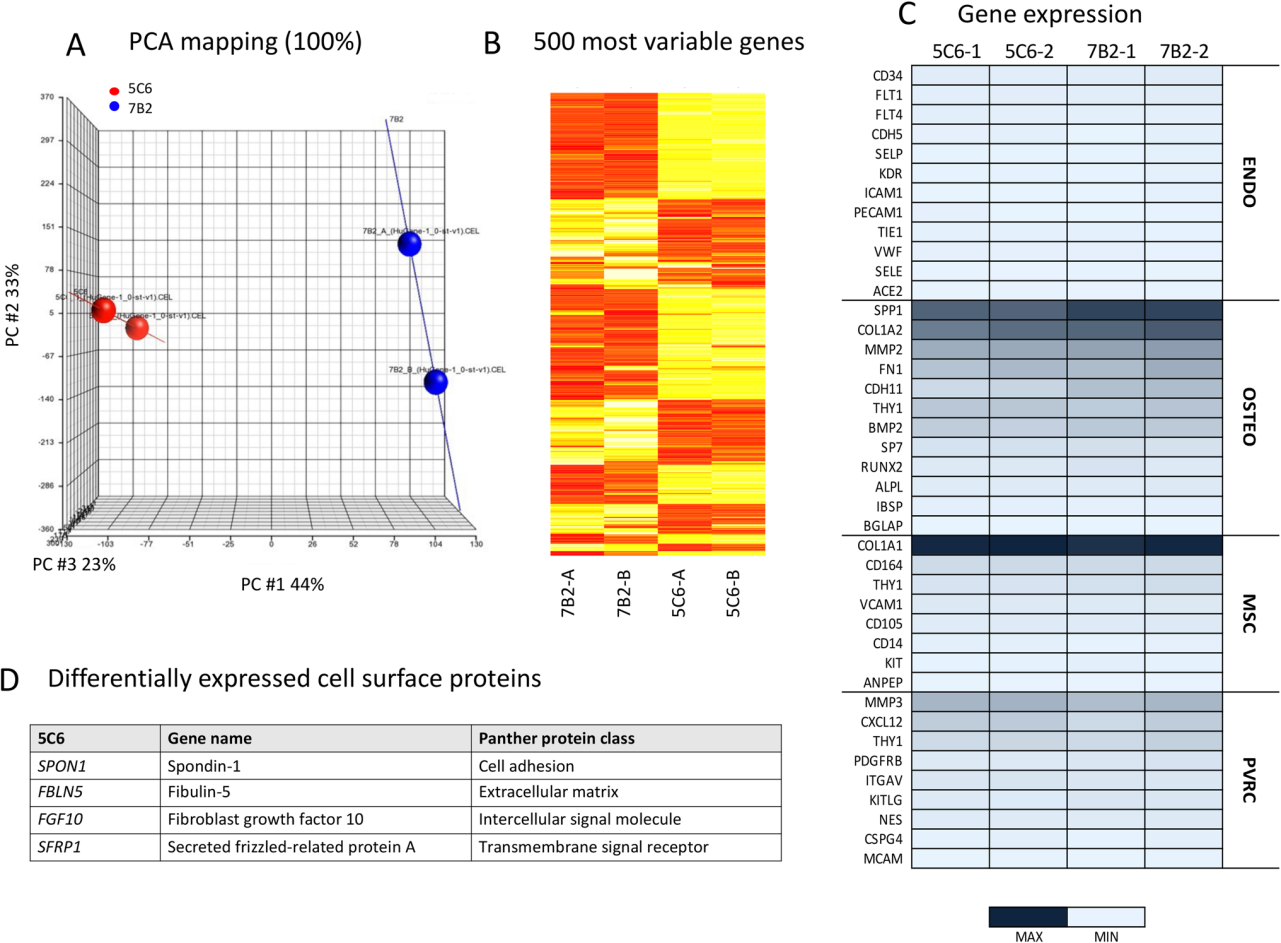
Gene profiling to characterise splenic stromal lines. Differential gene expression analysis was performed on stromal clones hu5C6 and hu7B2 which differ in capacity to support myelopoiesis in the human bone marrow. Transcriptome analysis was performed in duplicate using Affymetrix Human Gene 1.0ST GeneChips®. (***A)*** Principal components analysis (PCA) showed variability in gene expression for each line. (***B)*** Hierarchical clustering identified several main clusters amongst the top 500 most variable genes. (***C)*** Data mining was used to plot signal values for genes specific for endothelial cells (ENDO), osteogenic cells (OSTEO), mesenchymal stem cells (MSC) and perivascular reticular cells (PVRC). (***D)*** Panther pathway analysis was used to classify the protein class of the 50 most variable genes and to identify cell surface and secreted proteins which are differentially expressed.

Transcriptome analysis using Affymetrix Human Gene 1.0ST GeneChips® was used to investigate the lineage origin of human stromal lines which support hematopoiesis. The analysis compared the hu7B2 and the hu5C6 lines which were found to differ in capacity to support myelopoiesis in the human bone marrow (BM) (Fig. 3). Distinct differences existed between these two lines in terms of gene expression indicated by principal components analysis (PCA). For example, variability in gene expression for the first principal component was 44% (Fig. 5*A*). One hypothesis is that these two lines reflect distinct lineages or cell types within a lineage. The alternate hypothesis is that differences are due to natural variation developing after in vitro culture. Hierarchical clustering revealed variability reflected in 4 main clusters which are also evident in heat maps depicting the expression of the 500 most variable genes (Fig. 5*B*). Despite this variability, data mining revealed a common lineage origin for each of the two lines (Fig. 5*C*). This involved retrieval of signal values for sets of genes selected from the literature related to lineage and function of endothelial cells, osteogenic cells, mesenchymal stem cells and perivascular reticular cells. The most commonly expressed genes were related to osteogenesis like *SPP1, COL1A2, MMP2, FN1, CDH11, CD90 (THY1)* and *BMP2*, as well as genes expressed by perivascular reticular cells including *MMP3, CXCL12* and *THY1*, with no expression of genes specific to endothelial cells or to mesenchymal stem cells except *COL1A1*, which is also a marker of osteogenic cells (Fig. 5*C*). These data show that both these two human spleen stromal cells are reflective of perivascular or perisinusoidal reticular cells with osteogenic differentiation potential. Similar lineage findings were made with murine spleen stromal lines which support myelopoiesis (O’Neill *et al*. 2019).

Genes differentially expressed by the two lines were then identified through the selection of the 50 most variable genes, Panther Pathway analysis to identify Protein Class and then selection of genes which encode cell surface molecules. Only genes expressed by hu5C6 were identified under these criteria and these are shown in Fig. 5*D*. hu5C6 did not support hematopoiesis in overlaid human bone marrow progenitors, although it did support myelopoiesis in overlaid murine bone marrow progenitors (data not shown). It also reflects a perivascular reticular cell type which is an important cellular component of HSC niches in bone marrow and spleen. Three of the four genes identified are known regulators of the WNT/β-catenin signalling pathway which is crucial for HSC self-renewal (Luis *et al*. 2009), and is a powerful regulator of hematopoieis in murine spleen co-cultures (Lim *et al*. 2018). These genes encode proteins which inhibit or modulate the WNT/β-catenin signalling pathway and could maintain HSC in a state of quiescence, so limiting their differentiation to myeloid cells. *FBLN5* encodes an inhibitor of the WNT/β-catenin pathway and is a suppressor of cell invasion (Chen *et al*. 2015), while *SFRP1* is highly expressed by stromal cells which maintain HSC and supports homeostasis through extrinsic inhibition of β-catenin. (Renstrom *et al*. 2009). Finally, *FGF10* encodes a key regulator of signalling for cell differentiation from stem cells and is a modulator or inhibitor of WNT signalling (Watson and Francavilla 2018). *SPON1* encodes Spondin-1 an extracellular matrix protein with a role in neuronal regeneration, but is not known to regulate hematopoiesis (Woo *et al*. 2008). Indeed, hu5C6 appears to reflect a hematopoietic niche although it differs from hu7B2 in ways unrelated to cell lineage, and more in terms of the ability to regulate or specifically inhibit HSPC proliferation and differentiation.

## Discussion

Human splenic stromal cell lines have been isolated from long-term spleen cultures and characterised in terms of their hematopoietic support capacity for myeloid and dendritic lineage cells in line with previous studies in mice (Despars *et al*. 2004; Periasamy *et al*. 2018; O’Neill *et al*. 2019). Indeed, this represents a first report of human stromal lines reflecting perivascular or perisinusoidal reticular cells which provide hematopoietic support for HSPC from the human bone marrow (Corselli *et al*. 2013) and spleen (Inra *et al*. 2015; Oda *et al*. 2018). Human splenic LTC can be readily established and maintain the production of myeloid cells for an extended period as demonstrated previously in the mouse model (Periasamy *et al*. 2009). Stromal cells can be isolated, cloned and expanded to achieve multiple independent lines. Selected lines were shown to provide hematopoietic support capacity for overlays of human spleen progenitors, and then for progenitors from the human and murine bone marrow. To date, we have not attempted to derive stromal lines from the human bone marrow or cord tissue and to assess the capacity to support hematopoiesis. However, previously, we showed that stromal cell line derivation out of spleen long-term cultures was uniquely specific to spleen, and not to murine thymus, lymph node or blood (Ni and O’Neill 1998). Some long-term cultures of murine bone marrow did support the outgrowth of stroma (Ni and O’Neill 1998), but these did not support myelopoiesis, which appears to be a unique property of spleen tissue (Ni and O’Neill 2001).

The human spleen provides HSPC which can seed stroma and differentiate to give long-term production of a novel dendritic-like cell (L-DC), and short-term production of cDC-like cells and some myeloid DC (Fig. 2*C*). This result reflects the production of DC in human LTC (Fig. 1). It also reflects previous studies in mice which demonstrate the presence of HSPC in murine spleen which can self-renew in stromal co-cultures to continuously produce the novel subset called L-DC (Petvises and O’Neill 2014b). Human Lin^−^ BM is a rich source of HSPC and seeds human stromal lines like hu7B2 with the production of dendritic-like cells within 7 d (Fig. 3). In contrast to the human bone marrow and spleen, the human cord blood was found to be a very poor source of progenitors for seeding hu7B2 co-cultures (Fig. 4). Upon analysis of progenitor subsets, cord blood was shown to contain very few HSC and far higher numbers of MPP and CMP. The low representation of HSC amongst all progenitors in cord blood would appear to be the reason for the inability to sustain myelopoiesis past 14 to 21 d (Fig. 4*A*). The possibility however exists that cord tissue contains stromal cells which may support distinct cord blood hematopoietic progenitors or precursors, but this remains to be analysed.

In this study, it has been possible to compare human tissues as a source of hematopoietic progenitors/precursors. Human Lin^−^ spleen is an enriched source of HSPC. When co-cultured above the hu7B2 stromal line, a majority population of immature DC as hCD11c^−/lo^HLA-DR−hCD11b^−/lo^ cells was produced with only minor representation of HLA-DR^+^ cDC-like cells and myeloid or monocyte-derived DC. The immature DC subset appears to represent a counterpart cell to murine L-DC (O’Neill *et al*. 2004; Tan *et al*. 2011; Periasamy and O’Neill 2013). Variability in hCD11b expression has also been observed by others in human bone marrow co-cultures (Robinson *et al*. 1999), suggesting that hCD11b is a less reliable marker for identification of human DC in vitro compared with mice. Indeed, CD11b is expressed on a range of cell types including granulocytes, monocytes, DC, NK cells and some T and B cells (Stewart *et al*. 1995). hCD86 expression was also low and variable and so appears to be a more highly predictive marker for murine DC than human DC. While spleen co-cultures transiently support the production of a minority population of hCD11c^+^hCD11b^+^HLA-DR^+^hCD14^+^ cells resembling monocyte-derived DC (Kampgen *et al*. 1994; Romani *et al*. 1994; Sallusto and Lanzavecchia 1994; Caux *et al*. 1997, 1996), it is not yet known which precursors in human spleen give rise to these cells, or their relationship to the L-DC and cDC-like cells also produced in co-cultures.

Transcriptome analysis of two human splenic stromal lines addressed their lineage origin and their different hematopoietic support capacity. These cell lines do not appear to differ in lineage origin as stromal cells. Both the 7B2 and 5C6 lines express genes specific for perivascular reticular cells like *MMP3*, *THY1* and *CXCL12* (Fig. 5*C*). As with similar murine cell lines, human splenic stromal lines described here express genes reflective of osteogenesis. In the case of mice, stromal lines were also shown to have the potential to undergo osteogenic differentiation (O’Neill *et al*. 2019). This result is consistent with perivascular reticular cells as osteogenic progenitors, a phenotype which may relate to their hematopoietic support capacity.

Differential gene expression analysis identified several cell surface proteins expressed by hu5C6 but not by hu7B2 which are regulators of the WNT signalling pathway. This is significant in light of evidence that WNT signalling is essential for HSC self-renewal and maintenance within the niche and Wnt3a^−/−^ mice are severely compromised in hematopoiesis (Luis *et al*. 2009). The combined roles of proteins encoded by *FBLN5*, *FGF10* and *SFRP1* is to maintain homeostasis amongst HSC through negative regulation of the WNT/β-catenin signalling pathway, so inhibiting differentiation. Previous studies in this lab also identified a role for WNT signalling in in vitro hematopoiesis in co-cultures of murine splenic stroma (Lim *et al*. 2018). That study used specific pathway inhibitors to show in vitro hematopoiesis dependent on WNT and Notch signalling, as well as the CXCL12/CXCR4 and SCF/c-KIT receptor-ligand interactions (Lim *et al*. 2018). Subsequent addition of inhibitory antibodies and small molecule inhibitors to spleen stromal co-cultures confirmed CXCL12, CSF1, VCAM1 and SPP1 as potential hematopoietic regulators (Lim *et al*. 2018). These are known pathways and factors which regulate hematopoietic niches in bone marrow (Ogawa 2002; Sugiyama *et al*. 2006; Ding *et al*. 2012; Corselli *et al*. 2013). Gene profiling of murine splenic stromal cell lines which support in vitro myelopoiesis (Periasamy *et al*. 2018; O’Neill *et al*. 2019) also identified perivascular reticular lineage cells with the capacity to undergo osteogenic differentiation in vitro (O’Neill *et al*. 2019). Some murine stromal lines were shown to support ectopic myelopoiesis in vivo following engraftment under the kidney capsule of mice (O’Neill *et al*. 2019).

Combined studies in mice and humans now reinforce the role of mesenchymal perivascular/perisinusoidal reticular cells in extramedullary hematopoiesis, and implicate these cells as essential niches for myelopoiesis in spleen.

## Conclusions

Extramedullary hematopoiesis has been described for spleen although little knowledge exists for the role of spleen stromal cells as a niche supporting hematopoietic stem and progenitor cell differentiation. Here we describe a mesenchymal stromal cell type in the human spleen which supports hematopoiesis with the production of specific subsets of myeloid and dendritic cells unique to spleen. The human splenic stroma resembles murine stroma and represents a perivascular niche which supports myelopoiesis in spleen.

## Abbreviations

APC: Antigen-presenting cells
BM: Bone marrow
cDC: Conventional dendritic cells
CMP: Common myeloid progenitors
DMEM: Dulbecco’s minimal essential medium
FMOC: Fluorescence minus one controls
HSC: Hematopoietic stem cells
HSPC: Hematopoietic stem progenitor cells
LTC: Long-term cultures
L-DC: Dendritic-like cells produced in LTC
MPP: Multipotential progenitors
OVA: Ovalbumin
pDC: Plasmacytoid dendritic cells
sDMEM: Supplemented DMEM
